# The Expression of Pre- and Postcopulatory Sexually Selected Traits Reflects Levels of Dietary Stress in Guppies

**DOI:** 10.1371/journal.pone.0105856

**Published:** 2014-08-29

**Authors:** Md. Moshiur Rahman, Giovanni M. Turchini, Clelia Gasparini, Fernando Norambuena, Jonathan P. Evans

**Affiliations:** 1 Centre for Evolutionary Biology, School of Animal Biology, University of Western Australia, Crawley, Western Australia, Australia; 2 School of Life and Environmental Sciences, Faculty of Science, Engineering and Built Environment, Deakin University, Warrnambool Campus, Warrnambool, Victoria, Australia; Arizona State University, United States of America

## Abstract

Environmental and ecological conditions can shape the evolution of life history traits in many animals. Among such factors, food or nutrition availability can play an important evolutionary role in moderating an animal's life history traits, particularly sexually selected traits. Here, we test whether diet quantity and/or composition in the form of omega-3 long chain polyunsaturated fatty acids (here termed ‘n3LC’) influence the expression of pre- and postcopulatory traits in the guppy (*Poecilia reticulata*), a livebearing poeciliid fish. We assigned males haphazardly to one of two experimental diets supplemented with n3LC, and each of these diet treatments was further divided into two diet ‘quantity’ treatments. Our experimental design therefore explored the main and interacting effects of two factors (n3LC content and diet quantity) on the expression of precopulatory (sexual behaviour and sexual ornamentation, including the size, number and spectral properties of colour spots) and postcopulatory (the velocity, viability, number and length of sperm) sexually selected traits. Our study revealed that diet quantity had significant effects on most of the pre- and postcopulatory traits, while n3LC manipulation had a significant effect on sperm traits and in particular on sperm viability. Our analyses also revealed interacting effects of diet quantity and n3LC levels on courtship displays, and the area of orange and iridescent colour spots in the males’ colour patterns. We also confirmed that our dietary manipulations of n3LC resulted in the differential uptake of n3LC in body and testes tissues in the different n3LC groups. This study reveals the effects of diet quantity and n3LC on behavioural, ornamental and ejaculate traits in *P. reticulata* and underscores the likely role that diet plays in maintaining the high variability in these condition-dependent sexual traits.

## Introduction

The environmental and ecological conditions (biotic and abiotic) experienced by animals early in life can play a critical role in shaping life history traits and consequently individual fitness [1]. These traits and fitness consequences include growth [2], [3], survival [4], [5] and reproduction [6], [7], among others. Among the environmental factors contributing to these effects, dietary resources can play a pivotal role in shaping fitness by affecting an animal's physiology, behaviour, immunity and life history traits (e.g. [8], [9]) as well as the expression of sexually selected traits (e.g. [10]–[12]).

Sexually selected traits used by males to attract females and/or compete for access to mates are often thought to exhibit heightened condition dependence compared to non-sexual traits [13]–[15]. Accordingly, several studies have shown that males fed diets either of high quantity and/or quality exhibit enhanced ornamental (e.g. [16]–[19]) and behavioural traits (e.g. [18]–[22]) compared to males fed inferior diets. Dietary manipulation can also influence the expression of traits subject to postcopulatory sexual selection, such as sperm motility [23], [24], the proportion of live sperm in the ejaculate (sperm viability) (e.g. [18]), the number of sperm produced (e.g. [25]), sperm length (e.g. [19]) and testes size [26]. These latter findings are highly relevant for species where females mate with multiple males within a single reproductive episode (polyandry), where sperm competition (where ejaculates from different males compete for fertilizations [27]) and cryptic female choice (where females influence this competition [28], [29]) extend the opportunities for intra- and intersexual selection beyond mating. In both pre- and post-copulatory episodes of sexual selection, male secondary sexual characteristics may function as ‘honest’ indicators of male quality during mating contest/sperm competition and (cryptic) female choice [30]–[32].

Polyunsaturated fatty acids (PUFAs) are key nutrients moderating animal development and growth and underpin numerous critical physiological processes, including reproduction, vision, and brain development [33]–[35]. Animals cannot synthesize PUFAs *de novo* which must be obtained from the diet. PUFAs can therefore contribute to the condition-dependent expression of sexual traits, and more generally the behavioural characteristics of many species (e.g. [36]–[38]). PUFAs may also play a critical role in the development of fertility traits, particularly in determining the structural properties of spermatozoa. In most vertebrates, sperm comprise high levels of long-chain (LC) PUFAs (polyunsaturated fatty acid with 20 or more carbon atoms), as revealed by studies of spermatozoa in humans [39], birds [40] and a variety of livestock [41]. In these groups, PUFAs serve as structural determinants of gamete membranes [42] and play an essential role in gametogenesis [43] by supplying energy for gonad maturation [44]. They also influence membrane fluidity and mobility [45] and the fusion capacity of spermatozoa [46], [47]. Despite the importance of PUFAs in regulating behaviour and fertility traits, their role in determining the expression of traits subject to pre- and postcopulatory sexual selection is largely unknown.

The guppy *Poecilia reticulata* offers a useful model for investigating the functional co-dependence of pre- and postcopulatory traits on dietary resources. Guppies are polyandrous livebearing fish in which males and females exhibit marked sexual dimorphism. Males exhibit complex colour patterns composed of orange (carotenoid and pteridine), iridescent (structural), and black (melanin) spots, most of which play a role in sexual selection in this species [48]. For their part, females express cryptic coloration and exhibit consistent sexual preferences towards males exhibiting relatively large and bright colour spots, particularly orange, which males advertize to females during ritualized courtship displays [48]. Importantly, many male ornamental and behavioural traits are sensitive to environmental factors, particularly resource availability [49]–[51], and studies have shown that males in good condition exhibit enhanced pre- and postcopulatory sexual traits [52], [53]. In particular, two recent studies on guppies have tested whether the expression of pre- and postcopulatory sexual traits is influenced by dietary resource availability. In an initial study, Devigili et al. [18] reported that both precopulatory (behaviour and sexual coloration) and postcopulatory (sperm viability) traits were impacted by dietary restriction, with males fed restricted diets exhibiting reductions in each of these traits. Subsequent work that tested for interacting effects of dietary carotenoid levels (quality) and dietary restriction (quantity) [19] revealed no evidence that carotenoid levels influenced either pre- or postcopulatory sexual traits, although Devigili et al.'s [18] finding that dietary limitation had deleterious effects on all of these traits was corroborated.

In the present study we extend previous studies of condition dependence in guppies to determine whether the experimental manipulation of dietary omega-3 long chain polyunsaturated fatty acids (n3LC) levels, diet quantity, and their potentially interacting effects, influence the expression of precopulatory (sexual behaviour and colour patterns) and postcopulatory (velocity, viability, number and length of sperm) traits. Since environmental conditions experienced during an individual's lifetime can influence numerous life history traits (e.g. [54]–[56]), we manipulated diet quantity and n3LC levels from the onset of sexual maturity (around 13 weeks) until males were aged six months. In our study we ensured that n3LC levels were set at minimal levels in the restricted group, thus maximising any impact of n3LC deficiency on the observed traits. Because the expression of male sexual traits is sensitive to diet quantity [18], [19], we explore the effects of n3LC manipulation under experimentally low and high food levels, thus potentially exposing any interactive effects of diet quantity and n3LC levels on the expression of pre- and postcopulatory sexual traits.

## Materials and Methods

### Ethics Statement

The measures of precopulatory traits were performed in conditions that mimic natural conditions. For postcopulatory traits analysis, fish were anaesthetised to render them immotile during procedures (e.g. sperm extraction, photograph). This study was carried out in accordance with the Australian Code of Practice for the Care and Use of Animals for Scientific Purposes. This work was carried out under University of Western Australia's Animal Ethics approval RA/3/100/513.

### Animals and Dietary Treatments

The guppies used in this experiment were reared from descendants of fish captured from a natural population in Queensland (2006) and maintained following the same protocols as described in Rahman et al. [19]. One hundred and twenty males were used for the experiment. Males, which were aged three months at the start of the trials, were assigned haphazardly to one of two experimental diet treatments that differed in the level of omega-3 long-chain polyunsaturated fatty acids (hereafter termed ‘n3LC-enriched’ and ‘n3LC-reduced’; *n* = 60 per treatment). Each of these diet treatments was further divided haphazardly into two diet quantity treatments corresponding to overall feed levels (‘High-Quantity’ and ‘Low-Quantity’). Our experimental design therefore explored the main and interacting effects of two factors on male traits, with *n* = 30 fish assigned to each of the four treatments. The two experimental diets (n3LC-enriched and n3LC-reduced) were formulated to contain 45% protein and 10% lipid, thus differing only in the composition of the added lipid [57]. These diets were manufactured as steam pelleted feed following previously described procedures [58], and then ground and sieved to a particle size of 100–250 µm. Both diets were identical in composition, with the exception of the oil blend used as added fat (Tables 1 and 2). Specifically, the two blends (Table 2) were formulated so that they contained similar levels of saturated fatty acids (SFA) and n-6 polyunsaturated fatty acids, but differed in their n3LC content (high [12.9%] in the n3LC-enriched diet and only a trace amount [1.8%] in the n3LC-reduced diet), which was balanced off by increasing amounts of monounsaturated fatty acids (MUFA) in the n3LC-reduced diet (see also Online Supplementary Material; Table S1). The trace amount of long-chain polyunsaturated fatty acids (LC-PUFA), primarily in the form of 22∶6n-3, in the n3LC-reduced diet came from the fishmeal used as a protein source in both diets (fishmeal is used to provide protein and ensure palatability but also contains small amounts of LC-PUFA). Once assigned to their allocated diet treatments, males were reared individually in separate 2L aquaria (illuminated on a 12∶12 h cycle with Philips TLD 36 W fluorescent lamps) for a period of three months, and fed standard amounts of the crumbled diet once daily (six days per week) at a rate of 4% (1.9 mg; High-Quantity) or 1% (0.5 mg; Low-Quantity) of their body weight until they were six months old. Male guppies exhibit largely determinate growth from approximately 13 weeks (the onset of sexual maturity), and thus diet levels could be fixed at a standard quantity throughout the treatment phase.

**Table 1:** Ingredients used to formulate the experimental diets.

Ingredients (g/kg)	Enriched	Reduced
Pregel starch	218.72	218.72
Blood meal	78.58	78.58
Fish meal	65.48	65.48
Poultry by product meal	183.36	183.36
Soy protein concentrate	196.45	196.45
Whey protein concentrate	65.48	65.48
Lupin	130.97	130.97
Minerals & vitamins	5.00	5.00
Choline	3.00	3.00
Methionine	3.00	3.00
Lysine	1.50	1.50
Oil blend "High"	48.46	-
Oil blend "No"	-	48.46

**Table 2:** Formulation of oil blends in the diets.

Oil blends (%)	n3LC-enriched	n3LC-reduced
Cod Liver Oil	45.0	-
Fish Oil (Anchovy)	45.0	-
Soybean oil	10.0	1.8
High oleic sunflower oil	-	55.0
Palm oil	-	36.7
Linseed oil	-	6.5

### Measurements of Sexually Selected Traits

#### Precopulatory traits

The methods used to measure precopulatory traits followed those described previously by Rahman et al. [19]. Briefly, mating behaviour trials took place between 08.00 and 12.00 to correspond with the peak of sexual activity in this species [48]. For these trials, we used an 8L observation tank for each male (28.5×14.5×19 cm, filled to 14 cm) containing aquarium gravel and artificial pondweed. In each trial, a non-virgin female from a mixed-sex (stock) aquarium was placed in the tank and allowed to settle overnight. Females were approximately matched for size (by eye) across trials and used only once. The following day, a single male from one of the treatment groups was placed in the aquarium and allowed to settle for at least five minutes or until he showed sexual interest in the female (i.e. following the female or engaging in courtship). For each 10 minute trial, we recorded male mating behaviour as the number of sigmoid displays (males arch their body in a characteristic s-shaped posture and quiver), gonopodial thrusts (forced mating attempts in which males approach females from behind and attempt copulations without prior courtship or female solicitation), and the time (in seconds) spent by the male courting or chasing the female (a measure of the male's overall sexual interest in the female, hereafter ‘sexual interest’). After the trial, each male was returned to its individual tank and maintained on the same diet treatment for a further seven days before being used for the body size, colour patterns, sperm traits and fatty acids analyses. This period of isolation after the mating behaviour trials ensured that males would have fully replenished their sperm supplies prior to measuring sperm number and carrying out sperm analyses (see below) [59], [60].

We measured the area of carotenoid and pteridine pigments (orange and yellow spots, hereafter summed as ‘orange’) and structural colours (blue, green, purple, silver and white, collectively termed ‘iridescence’) from digital photographs taken of each anaesthetized fish using *ImageJ* software (ImageJ website. Available: http://rsbweb.nih.gov/ij/. Accessed 4 August 2014). We also measured male body length (‘standard length’  =  distance in mm from the snout to the tip of the caudal peduncle; hereafter SL). The total number of orange and iridescent spots was also recorded as an index of colour complexity [61], [62]. The reflectance of each male's coloured spots was measured from the digital photographs using *ColourWorker* software (Chrometics Ltd. Website. Available: http://www.chrometrics.com. Accessed 4 August 2014; see details [19]). Briefly, each male was photographed along with a simulated Gretag Macbeth Standard with known reflectance to provide a reference for the software, allowing us to account for minor variation in lighting conditions among images. The raw uncompressed images (.NEF) were converted to TIFF files before being imported into ColourWorker. Once reflectance data were obtained, we used principal component analysis (PCA) to extrapolate PCs for the orange and iridescent spectra separately. Similar to previous studies, the first PC explained >87% of the total variability in orange spots, and >77% of the total variability in iridescent spots and represents mean spectral reflectance (typically correlated with brightness). We therefore used the first PC in our analysis [63].

#### Postcopulatory traits

Immediately after the digital photography, the anesthetized males were carefully dried and placed on a glass slide under a dissecting microscope with their gonopodium (intromittent organ) swung forward. A micropipette was used to add 500 µl of an extender medium (207 mM NaCl, 5.4 mM KCl, 1.3 mM CaCl_2_, 0.49 mMMgCl_2_, 0.41 mMMgSO_4_, 10 mM Tris, pH 7.5) at the base of the gonopodium. The use of the extender medium ensured that sperm bundles remained intact and quiescent until they were used for the sperm velocity assays [64]. Light pressure was then applied to each male's abdomen to expel all strippable sperm into the extender medium [65]. From this total sperm pool, we placed 2–3 spermatozeugmata (unencapsulated sperm bundles) for sperm velocity estimation in a single well of a 12-well multitest slide (MP Biomedicals, Aurora, OH, USA) coated with 1% polyvinyl alcohol (Sigma-Aldrich, Australia) to avoid sperm sticking to the glass slide [66]. We also extracted ten spermatozeugmata for sperm viability assays and the remaining sperm bundles were collected in a known volume of saline solution with 1% formalin (to prevent sperm degradation) and stored at 4°C until sperm number and length were measured (see below).

We used computer-assisted sperm analyses (CASA) to estimate sperm velocity using the CEROS sperm tracker (Hamilton-Thorne Research, Beverly, MA, USA). Sperm velocity was estimated immediately following activation by 150 mM KCl with 2 mg/L bovine serum albumin (BSA) [67]. The measures were based on an average of 85.0±4.65 SE sperm tracks per sample (mean value is taken for *n* = 112 males; *n* = 8 males did not produce sperm). As the average path velocity (VAP) was highly correlated with the straight line velocity (VSL) (r<0.95; P<0.0001) and also with the curvilinear velocity (VCL) (r<0.88; P<0.0001), we restricted our analysis of sperm velocity to VAP for brevity (note that results were unchanged whatever measure we used). The threshold value for defining static cells was predetermined at 24.9 µm/s for VAP [68].

A live/dead sperm viability assay (Invitrogen, Molecular Probes) was used to estimate the proportion of live sperm in the reserved sub-sample of each male's stripped ejaculate (following the same methods explained in [19]). The assay stains live sperm green with the membrane-permanent nucleic acid stain SYBR-14, and dead sperm red with propidium iodide, which penetrates damaged sperm cell membranes. The proportion of live sperm in each sample was then estimated from 200 sperm cells per sample.

Sperm number was estimated for the reserved portion of each male's stripped ejaculate using an improved Neubauer haemocytometer under 40× magnification (Leica DM1000 microscope) after vortexing each sample for 30 seconds. The average of five counts per male was used to estimate the total number of sperm in each stripped ejaculate [65]. Sperm number was corrected to allow for sperm that had been removed from each sample for the CASA and viability assays.

To estimate sperm length (distance in µm from the top of the sperm head to the tip of the flagellum), photographs of each male's sperm were obtained with an objective magnification of 40× (Leica DM1000 microscope) using a digital camera (Leica DFC320). Where possible, 20 undamaged spermatozoa were measured per male (mean number of sperm cells analysed per male = 19.73±0.15 SE; range = 10–20). *ImageJ* software was used to measure the total length of each sperm cell.

### Fatty Acids Content in Diets and Fish Tissue Samples

The experimental diets and the testes and body samples of the High-Quantity dietary groups were collected and preserved at -80°C for subsequent fatty acid analysis. After lipid extraction [69], a known amount of tricosanoate (23∶0) was added as internal standard, and fatty acids were esterified into methyl esters using the acid catalysed methylation method. The identification of FA methyl esters was determined using an Agilent Technologies GC 7890A gas chromatograph (Agilent Technologies, USA) equipped with an BPX70 capillary column (120 m, 0.25 mm internal diameter, 0.25 µm film thickness; SGE Analytical Science Pty Ltd, Ringwood, Vic, Australia), a flame ionisation detector (FID), an Agilent Technologies 7693 autosampler injector, and a split injection system, using chromatographic conditions previously reported [70]. Each of the fatty acids was identified relative to known external standards (Sigma-Aldrich, Inc., St. Louis, MO, USA, and Nu-Chek Prep, Elysian, MN, USA), and the resulting peaks were then corrected by the theoretical relative FID response factors and quantified relative to the internal standard.

### Statistical Analysis

No mortality was recorded during the experiment. Sperm number and sperm viability data were appropriately transformed prior to analysis (cube root transformation for sperm number and arcsine square root transformation for sperm viability). All analyses were performed using ‘R’ version 3.1.0 [71]. We calculated descriptive statistics (means, SEs, ranges etc.) using the ‘pysch’ package, tested for normality with the ‘nortest’ package, and carried out principal components analysis with the ‘FactoMineR’ package.

We performed two multivariate analyses of variance (MANOVA) to test for an overall effect of the two diet treatments (n3LC and diet quantity) and their interaction on (a) colour patterns (orange spot number, orange area, orange PC, iridescent spot number, iridescent area, iridescent PC) and (b) sperm traits (sperm swimming velocity, sperm viability, sperm number, total sperm length). Note that behavioural traits were excluded from the first MANOVA model because their distributions did not meet the assumptions of the multivariate test (see above). In both models, the treatment groups and their interaction were fitted as fixed effects, while male body length (standard length) was included as a covariate only in the first model since colour patterns were correlated with body size (see Results). As both models revealed an overall significant effect of one or both treatments on the traits of interest (see below), subsequent univariate analyses of variance (ANOVA) models were run to identify where these effects lie.

Next we used analyses of variance to compare the composition of fatty acids in the fish tissue samples (body and testes) for the two High-Quantity diet treatments (i.e. n3LC-enriched and n3LC-reduced; *n* = 30 tissue samples per treatment). Initially we used MANOVA to test for overall effects of diets on body and testes samples. We then used a series of one-way ANOVA models (reported in the Supplementary Material) to highlight where specific differences in fatty acid composition in diets and the body and testes samples of two High-Quantity groups lie. We used ‘type II’ tests in all models as these offer more powerful approaches for estimating sums of squares in the absence of interactions which were largely absent or weak in our analysis (as in our analysis; see [72]). All analyses of variance models were run using the ‘car’ package of ‘R’.

## Results

Overall, the High-Quantity group males were significantly larger than those assigned to the Low-Quantity dietary group (SL mean ± SE; High-Quantity: 16.61±0.14 mm, Low-Quantity: 14.89±0.09 mm; F_1, 113_ = 113.5, P<0.001).

### Effect of Diet on Pre- and Postcopulatory Traits

**Figure 1:**
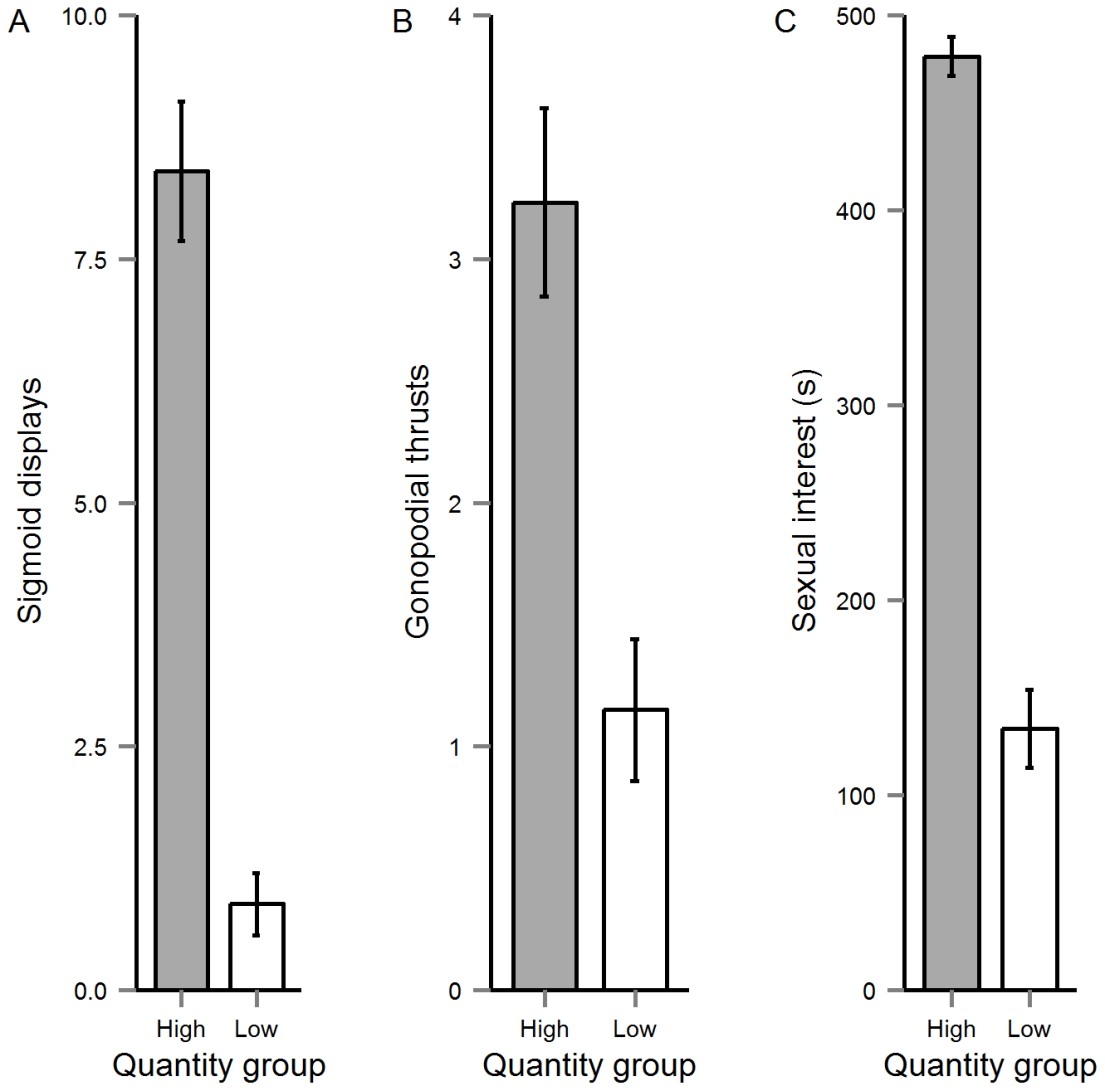
The mean (±SE) effect of diet quantity on (A) sigmoid displays, (B) gonopodial thrusts and (C) sexual interest.

The two MANOVA models revealed overall significant main effects of one or both of the diet treatments on male colour patterns and sperm traits (see Table 3). Our separate GLMM models also revealed significant effects of treatment on the behavioural traits. Specifically, these latter models revealed that diet quantity had a significant effect on all male sexual behaviours (sexual interest, sigmoid displays and gonopodial thrusts) and a marginal interactive effect of both treatments on sigmoid displays (see Online Supplementary Material; Table S2). By contrast, we detected no significant effect of n3LC on any of the precopulatory traits (see Online Supplementary Material; Table S2). The analyses revealed that diet-restricted (Low-Quantity) males performed significantly fewer courtship (sigmoid) displays and gonopodial thrusts, and exhibited a reduction in sexual interest during the behavioural trials, compared with the High-Quantity group (Figures 1A, 1B and 1C).

**Table 3:** MANOVA results of diet quantity, n3LC and their interaction on male colour patterns and sperm traits.

Response	test stat	F-ratio	df	den df	P(>F)
***Colour patterns***					
Quantity	0.295	7.124	6	102	***<0.001***
n3LC	0.019	0.333	6	102	0.918
SL	0.394	11.04	6	102	***<0.001***
Quantity* n3LC	0.076	1.393	6	102	0.225
***Sperm traits***					
Quantity	0.522	27.02	4	99	***<0.001***
n3LC	0.163	4.808	4	99	***0.001***
Quantity* n3LC	0.055	1.439	4	99	0.227

Significant P-values are marked in bold and italic fonts.

**Figure 2:**
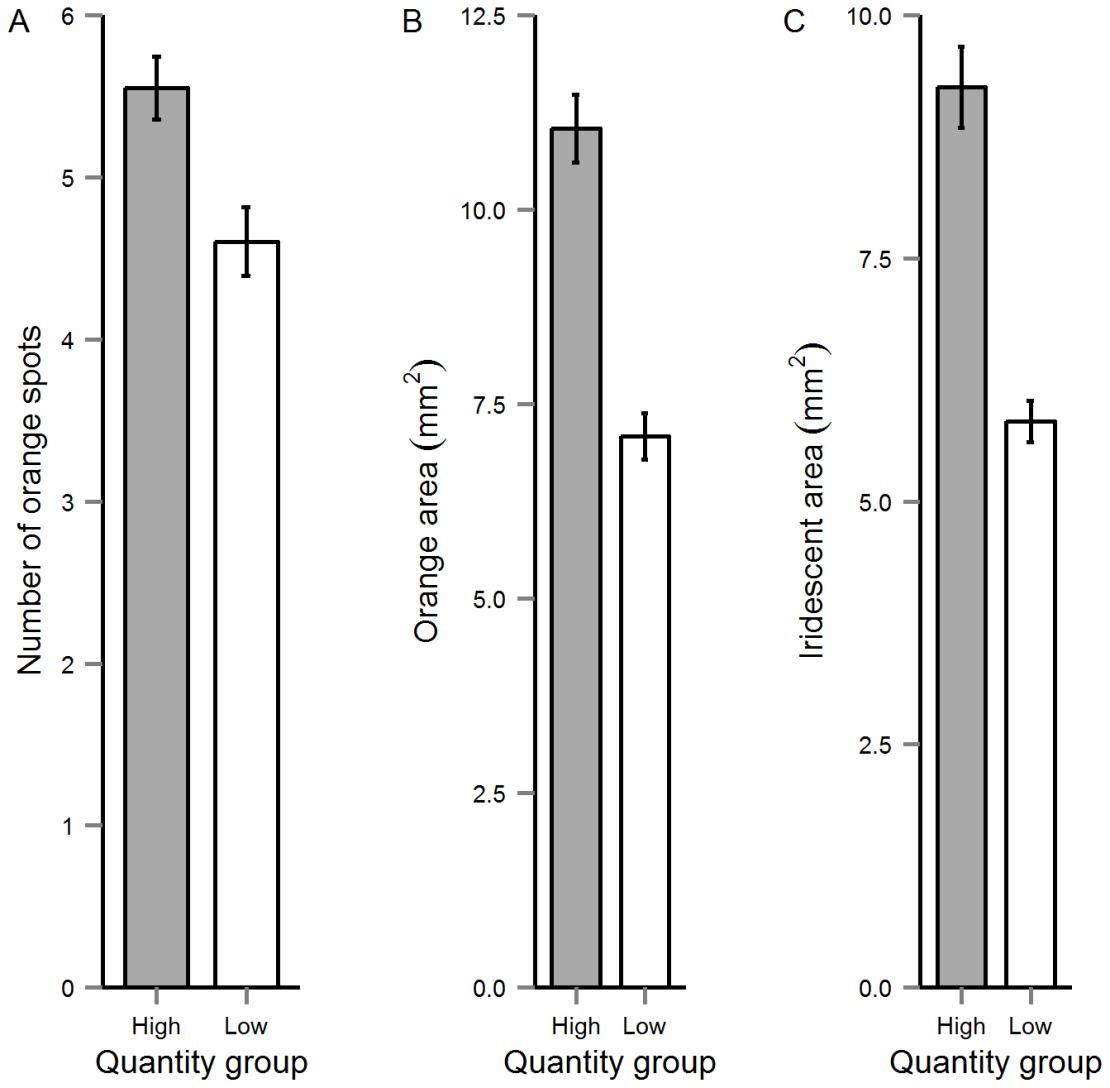
The mean (±SE) effect of diet quantity on (A) number of orange spots, (B) orange area and (C) iridescent area.

**Figure 3:**
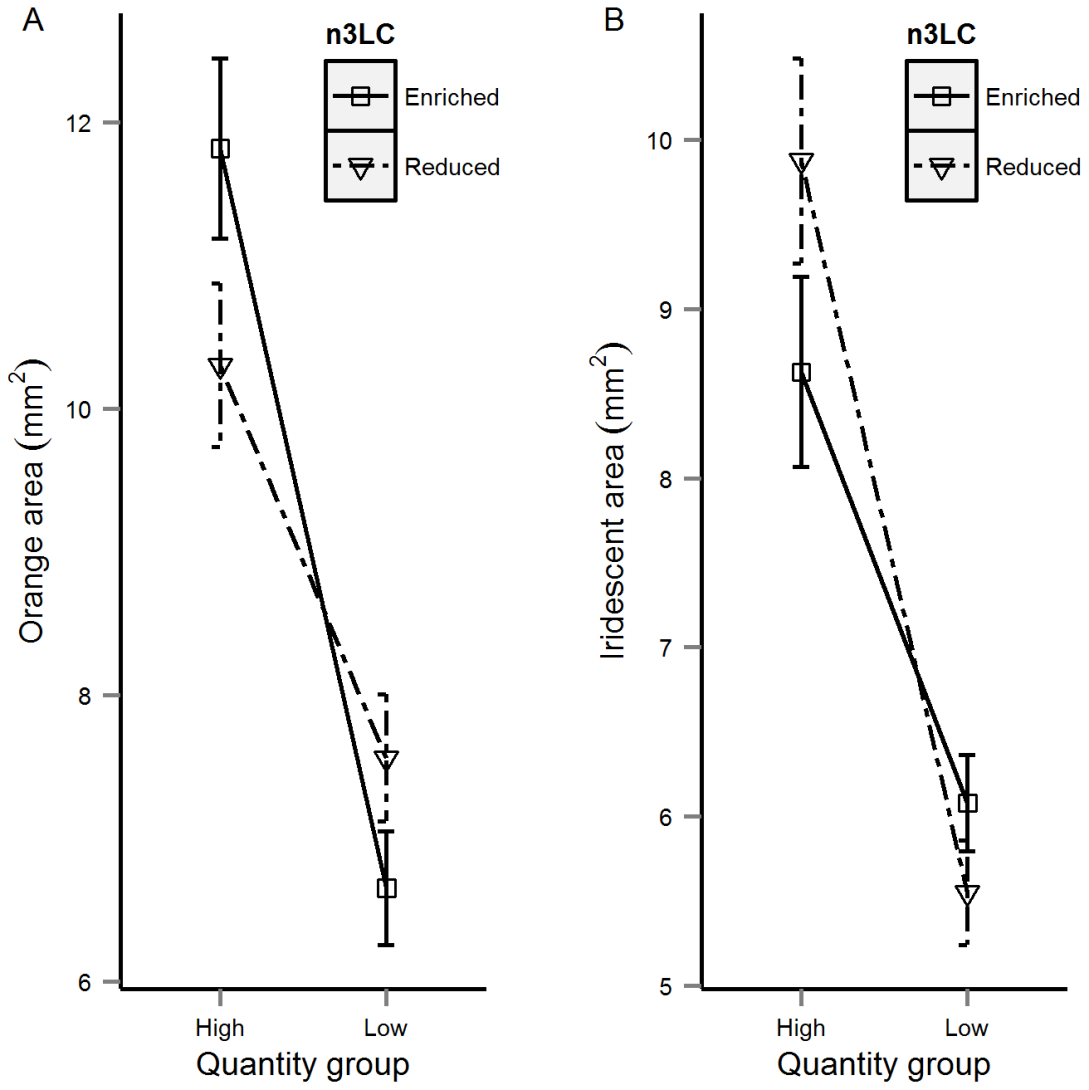
The quantity-by-n3LC interaction effect (mean ± SE) on (A) orange area and (C) iridescent area.

Our univariate analyses also revealed significant effects of diet quantity on the size, number and brightness of the males' colour spots (see Online Supplementary Material; Table S2). Specifically, males assigned to the Low-Quantity diet exhibited significantly fewer orange spots and a reduction in the area of orange and iridescent spots than those in the High-Quantity group (Figures 2A, 2B and 2C). We also detected significant interacting effects of quantity-by-n3LC on the area of orange and iridescent colour (see Figures 3A and 3B). The PC describing variation in the spectral characteristics of orange and iridescent spots [19], [73] varied between the diet quantity groups. Males assigned to the High-Quantity group had brighter iridescent spots than Low-Quantity males (Online Supplementary Material; Table S2).

**Figure 4:**
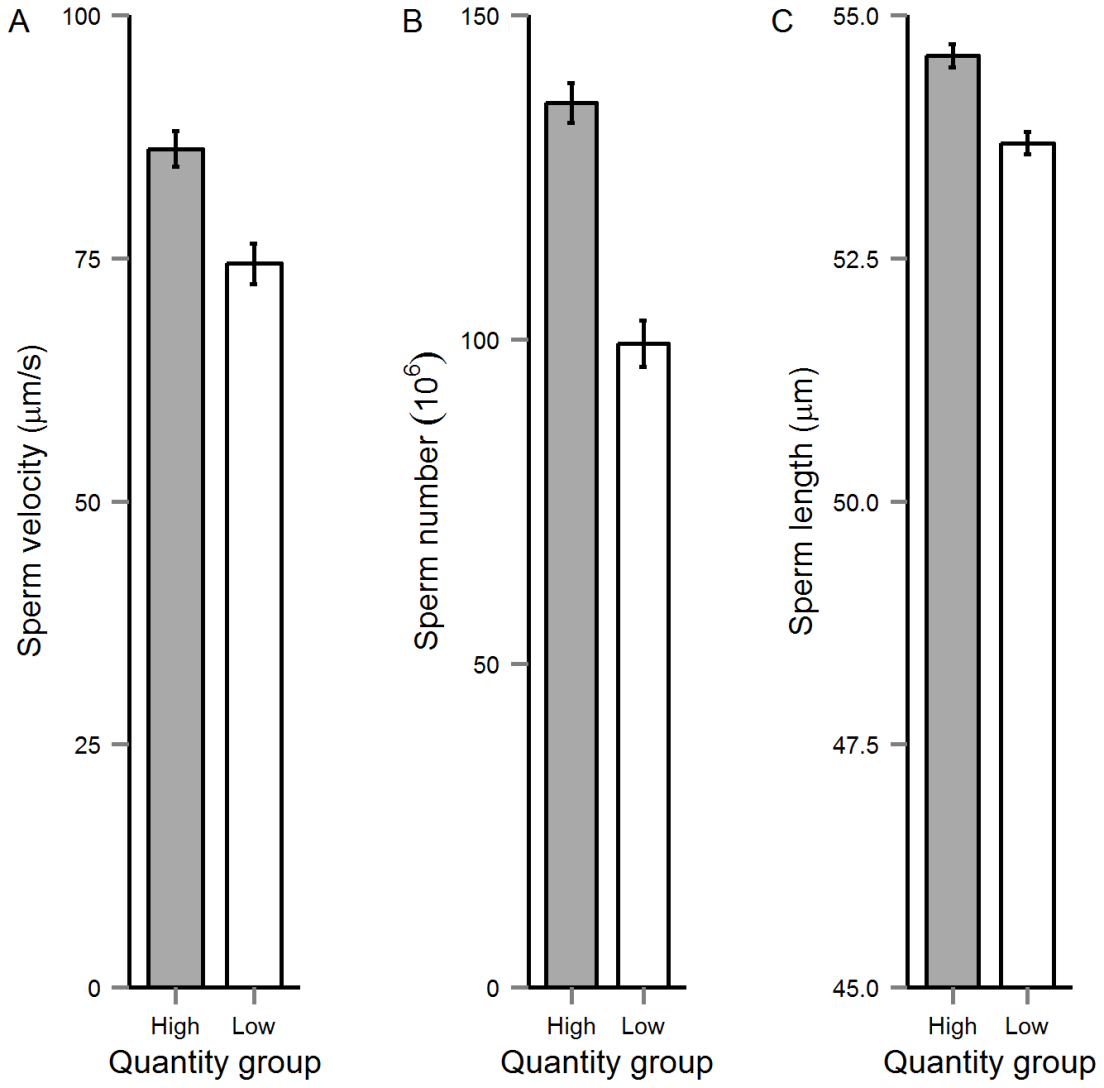
The mean (±SE) effect of diet quantity on (A) sperm swimming velocity, (B) sperm number and (C) total sperm length.

**Figure 5:**
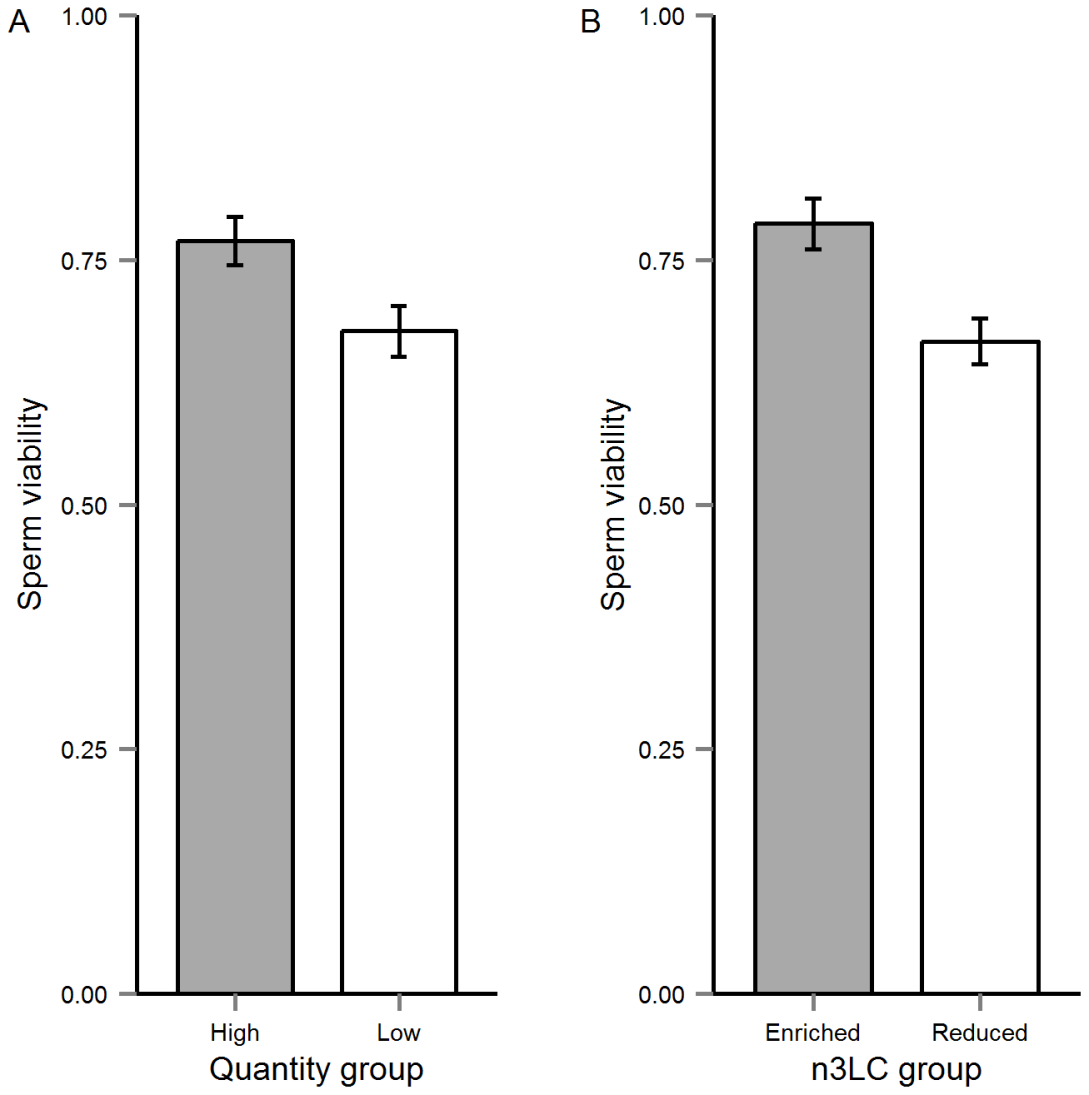
The mean (±SE) effect of (A) diet quantity and (B) n3LC on sperm viability.

The multivariate model describing treatment effects on sperm traits revealed significant effects of both diet quantity and n3LC levels (see Table 3). Our subsequent univariate models revealed that sperm swimming velocity, sperm viability, sperm number and sperm length were affected by diet quantity, while n3LC had a significant effect only on sperm viability (Online Supplementary Material; Table S2). The Low-Quantity males had slower-swimming, fewer, shorter and reduced number of viable sperm than their well-fed counterparts (see Figures 4A, 4B, 4C and 5A), whereas n3LC-reduced males had less viable sperm than n3LC-enriched group (Figure 5B).

### Fatty Acids Analyses

Our MANOVA model revealed that diet had significant effects on the accumulation of fatty acids in body and testes tissues (see Table 4). The subsequent univariate ANOVA models revealed that males fed n3LC-enriched diet had a significantly higher percentage of 14∶0, 16∶1n-7, 18∶2n-6, 20∶5n-3, 22∶5n-3, 22∶6n-3, saturated fatty acids (SFA), n-3 polyunsaturated fatty acids (n-3 PUFA), n-3-long chain polyunsaturated fatty acids (n-3-LC PUFA) than males fed n3LC-reduced diets, while the males fed n3LC-reduced diets had significantly higher content of 18∶1n-9 and monounsaturated fatty acids (MUFA) in their body tissues than their counterparts fed n3LC-enriched diets. In testes tissues, males assigned to n3LC-enriched diet had significantly more 20∶5n-3 and 22∶5n-3 than the n3LC-reduced group, while males fed n3LC-reduced diets had only significant amount of 18∶1n-9 than the n3LC-enriched group (see also Online Supplementary Material; Table S3).

**Table 4:** MANOVA results for fatty acids content (%) in body samples and testes tissues after the treatment.

Samples	Response	test stat	F-ratio	df	den df	P(>F)
Body	Fatty acids	0.88	10.8	24	35	***<0.001***
Testes	Fatty acids	0.76	4.05	24	31	***<0.001***

Significant P-values are marked in bold and italic fonts.

## Discussion

Our results reveal that dietary manipulations had a significant effect on the expression of pre- and postcopulatory sexually selected traits in male guppies. In the case of precopulatory sexually selected traits, we found that males assigned to the Low-Quantity group (fed a restricted diet) exhibited reductions in courtship (sigmoid) displays, gonopodial thrusts, sexual interest, area of orange spots and iridescent spots, and orange spot number compared to those assigned to the High-Quantity group. We also found significant effects of diet quantity and n3LC levels on ejaculate traits; Low-Quantity males produced slower-swimming, fewer, shorter and less viable sperm than those assigned to the *ad libitum* diet treatment, while n3LC-reduced had reduced number of viable sperm than n3LC-enriched males. Finally, we found that n3LC-enriched group accumulated significantly higher percentage of n3LC in their body and testes tissues than n3LC-reduced group. Below, we discuss each of these key findings in detail.

### Effect of Diet on Precopulatory Traits

Our results revealed significant effects of diet quantity on male sexual behaviour, thus supporting similar findings from guppies and other taxa showing that mating tactics and sexual interest can reflect a male's current nutritional condition [22], [50], [53], [74]. Indeed, our findings for the effect of diet quantity on male sexual behaviour are broadly similar to previous work on guppies, where males fed restricted diets performed reduced sigmoid displays compared to their well fed counterparts [18], [19]. We also found tentative evidence for interactive effects of diet quantity and n3LC levels on courtship behaviour, which suggests that the effects of diet quantity on this particular behaviour may be modulated by the quantity n3LC. Similar findings have been reported elsewhere, for example where dietary nutrients have significant effects on behavioural traits of males [22], [75], [76]. Interestingly, the present analyses also revealed that High-Quantity males performed significantly more gonopodial thrusts than those in the Low-Quantity group. This result was unanticipated in the light of previous research on the same population revealing no significant effect of dietary restriction on the frequency of gonopodial thrusts [18], [19]. However, the difference between studies may be due to slight variation in feeding levels, which in the case of Rahman *et al*.'s [19] study involved feeding fish at 4% body weight in the High-Quantity group (as we did) and 2% body weight in the Low-Quantity group (note that [18] did not specify weights and used a different diet [*Artemia*] to manipulate diet intake). In the present study, we set the Low-Quantity group at 1% body weight, thus potentially exacerbating nutritional stress and exposing condition dependence in a broader range of behaviours. Our results also revealed that the males' sexual interest was influenced by diet quantity, which is also consistent with other studies of the same species [18], [19], [53]. In particular, Low-Quantity males spent significantly less time following females than their counterparts assigned to the High-Quantity group. Notwithstanding minor difference among studies, overall our study complements previous work on guppies in revealing that male mating behaviour is strongly contingent on condition, and thus may provide honest signals of male quality during mate choice [50], [53], [77], [78].

As with the behavioural traits, we found that diet quantity had significant effects on the expression of the males' colour spots. Specifically, we found that diet quantity influenced the size of orange and iridescent spots, the total number of orange spots, and the spectral properties of iridescent spots. These findings largely corroborate previous studies on guppies [18], [19] and other species [79], [80] and suggest that, as with the behavioural traits, colour spots convey ‘honest’ information about male condition, thus potentially explaining their evolutionary maintenance through sexual selection [30], [81]. By contrast, we found no effect of n3LC supplementation on male colouration, although there was evidence for interacting effects of diet quantity and n3LC supplementation on the area of orange and iridescent spots. These interactions are intriguing and may indicate that the expression of these colour patterns are modulated by n3LC in the diet. In our experiment, juvenile guppies (<3 months) were fed a diet of live *Artemia* prior to the experimental feeding trials. Thus, the experimental males would have assimilated these in their tissues prior to the treatments because *Artemia* comprise a rich source of carotenoids [82], [83]. As dietary lipid quantity plays a key role in the assimilation of dietary carotenoids [84]–[86], it is possible that in the case of orange spots, males fed a diet containing high n3LC levels may exhibit enhanced colouration due to the role that dietary lipids play in modulating the absorption, transport and metabolism of these previously ingested carotenoids ([87], [88], but see also [89]). Such effects clearly have the potential to expose the quantity-by-n3LC interactions for orange colour spots exposed by our study, although we currently lack an explanation for the equivalent effects observed for iridescent colour spots. Future studies would benefit by investigating the effect of dietary carotenoids and LC-PUFAs quantity from birth (see [52]).

### Effect of Diet on Postcopulatory Traits

Our results revealed that diet quantity had significant effects on sperm swimming velocity, sperm number, sperm length and sperm viability, while n3LC levels had a significant influence on sperm viability only. Our finding that sperm swimming velocity was strongly contingent on diet quantity is consistent with a previous study on the same guppy population [19]. Similar to our findings, Cerolini et al. [23] also reported that in chickens, food restricted broiler breeder males produced significantly fewer motile sperm than those fed *ad libitum*. The findings from these studies may be explained by the reduced energy content of diets fed to food-restricted males. Dietary energy levels can affect gonadotrophin secretion, which in turn regulates the production of spermatozoa in animals [90]–[92]. To explicitly test the effects of energy levels on ejaculate traits, Selvaraju et al. [24] conducted a study where rams were fed diets differing in energy levels. Their findings revealed that rams fed a low-energy diet exhibited a significant reduction in sperm motility compared to those fed a high-energy diet, possibly due to role that energy levels play in modulating insulin-like growth factor-I (IGF-I) and concomitant effects on sperm function [24]. Thus, the present results might be attributable to the High-Quantity group having comparatively higher energy levels available to stimulate hormones that control sperm physiology.

Like previous studies [18], [19] we also found significant effect of diet quantity on sperm viability, indicating that Low-Quantity males produced significantly fewer viable sperm than their counterparts in the High-Quantity group (Figure 5A). Our results also revealed that n3LC-reduced males had significantly reduced number of viable sperm than the n3LC-enriched group (Figures 5B). This latter result is also consistent with findings reported in other taxa. For example, Al-Daraji et al. [93] reported that in Japanese quail, males fed diets supplemented with fish oil (high in omega-3) produced significantly more live sperm than those fed diets supplemented with sunflower oil (low in omega-3). Similarly, in an experimental study on humans, Robbins et al. [94] showed that men whose diets were supplemented with walnuts (rich in PUFAs) experienced significant improvement in sperm motility and sperm viability than those who consumed diets without any walnuts. Thus, our findings add to the growing evidence that n3LC levels are critical determinants of sperm quality in vertebrates whilst underscoring how the condition-dependent expression of ejaculate traits can reflect differences in dietary composition rather than overall energy levels.

We also found that High-Quantity males produced significantly higher number of sperm than the Low-Quantity group, again supporting prior evidence from guppies (see also [19], [25]) and other taxa [11], [24], [95]–[97]. Dietary energy levels are critically important for the production of hormones that promote reproductive activity, including ejaculate production [90]–[92]. This may, in turn, explain why Low-Quantity males in our experiment were both smaller and produced fewer sperm than their counterparts in the High-Quantity group. In an experiment with another population of guppies, Gasparini et al. [25] found that diet restricted males (fed limited *Artemia*) showed significant reduction in sperm number. Parker and Thwaites [98] similarly reported a reduction in both body size and ejaculate volume in rams fed restricted diets (either 75% or 50% of the control maintenance diet), while Sexton et al. [99] found that in chickens, fully fed broiler breeder males produced ejaculates comprising higher sperm concentrations than feed-restricted males (see also [100]). However, unlike previous studies on other taxa [93], [101]–[103], we found no significant effect of n3LC on sperm number.

We detected a significant effect of diet quantity on total sperm length; Low-Quantity males had comparatively shorter sperm than their counterparts fed *ad libitum* (see also [19]). This finding suggests that sperm length, along with body size and sperm numbers, may be compromised by male condition, possibly reflecting a difference in energy content between the High- and Low-Quantity diets. Previous work on guppies has revealed positive phenotypic correlations between sperm length and/or sperm numbers and body size [104]–[106]. Our finding that males assigned to the High-Quantity group were significantly larger than those in the Low-Quantity group may help explain why larger males produce larger ejaculates comprising longer sperm, assuming that all three traits are similarly influenced by dietary energy levels. Although relatively few studies have explored sperm morphometry in this context, Immler et al. [107] found that in Gouldian finches, changes in stress and sex steroid hormone levels accompanying changes in the social environment influenced sperm length, thus similarly revealing plasticity in sperm length in response to different environmental conditions. However, unlike some studies on other taxa [93], [94], [108], we found no significant effect of n3LC on sperm length. One possible explanation for the lack of significant effects of n3LC on sperm number and length in our study may be that n3LC males may have relied on previously ingested fatty acids obtained prior to reaching sexual maturity (i.e. from *Artemia*). Another explanation may be that n3LC needs to be incorporated with a minimum level of carotenoids, essential amino acids or vitamins (A or E) to influence those traits [102], [109]. These are clearly fruitful areas for further investigation.

### Fatty Acids Analyses

Many studies have revealed that fatty acid profiles in tissues, blood cells and semen samples reflect an individual's dietary quantity [110]–[115]. Our results similarly show that males fed n3LC-enriched diets accumulated a higher percentage of PUFAs in the body and testes tissues, while the n3LC-reduced group contained significantly more MUFAs in these tissues. Thus our results reveal that dietary supplementation of PUFAs can increase their uptake in tissue levels, most likely in the testes, which might have an impact on ejaculate quality (see above).

## Conclusions

In conclusion, our results reveal that diet quantity has a number of significant effects on both pre- and postcopulatory traits in male guppies, while n3LC had a significant effect only on sperm viability and interacted with diet quantity to influence sigmoid displays and colour area. Our results also confirm that the uptake of n3LC in body and testes tissues was directly influenced by the manipulation of PUFAs in the experimental diets. We strongly advocate further experiments that manipulate omega-3/omega-6 ratios (e.g. [116], [117]) and explore the interactive effects of PUFAs with other dietary resources such as carotenoids [109], [118], essential amino acids [119], [120], and vitamin A or E [102], [121]. We anticipate that such experiments will further emphasise the key role that nutritional stress plays in shaping male reproductive traits and the complex interactions among dietary resources that underlie such effects.

## Supporting Information

Table S1 **Fatty acids composition of the experimental diets.**
(DOCX)

Table S2 **Univariate models for all sexually selected traits.**
(DOCX)

Table S3 **ANOVA results for fatty acids content (%) in body samples and testes tissues after the treatment.** Significant P values are highlighted in bold and italic fonts.
(DOCX)
