# Supplementary material for: The Expression of Pre- and Postcopulatory Sexually Selected Traits Reflects Levels of Dietary Stress in Guppies
Source: PLoS One. 2014 Aug 29;9(8):e105856. doi: 10.1371/journal.pone.0105856 (PMC4149491; doi:10.1371/journal.pone.0105856)
Supplement: Table S1 — Fatty acids composition of the experimental diets. (DOCX) [file pone.0105856.s001.docx]

**Table S1.** Fatty acids composition of the experimental diets

| Fatty acids | n-3 LC-enriched | n-3 LC-reduced |
| --- | --- | --- |
|  | (% total fatty acids) | |
| 12:0 | 0.1 | 0.1 |
| 14:0 | 3.8 | 1.0 |
| 14:1n-5 | 0.1 | 0.1 |
| 15:0 | 0.3 | 0.1 |
| 16:0 | 18.9 | 19.5 |
| 16:1n-7 | 5.7 | 2.2 |
| 18:0 | 4.4 | 4.4 |
| 18:1n-9 | 27.8 | 49.0 |
| 18:1n-7 | 2.7 | 1.5 |
| 18:2n-6 | 16.0 | 15.8 |
| 18:3n-6 | 0.2 | 0.1 |
| 18:3n-3 | 2.1 | 3.0 |
| 18:4n-3 | 1.0 | 0.2 |
| 20:0 | 0.2 | 0.3 |
| 20:1n-11 | 0.2 | 0.1 |
| 20:1n-9 | 1.3 | 0.3 |
| 20:2n-6 | 0.2 | 0.0 |
| 20:3n-6 | 0.1 | 0.0 |
| 20:4n-6 | 0.5 | 0.1 |
| 20:3n-3 | 0.1 | 0.0 |
| 20:4n-3 | 0.5 | 0.1 |
| 20:5n-3 | 6.7 | 1.0 |
| 22:0 | 0.2 | 0.3 |
| 22:1n-11 | 0.9 | 0.0 |
| 22:1n-9 | 0.3 | 0.1 |
| 22:2n-6 | 0.0 | 0.0 |
| 22:4n-6 | 0.2 | 0.0 |
| 22:3n-3 | 0.0 | 0.0 |
| 22:5n-3 | 1.2 | 0.2 |
| 24:0 | 0.1 | 0.2 |
| 22:6n-3 | 4.3 | 0.5 |
| TOT | 100.0 | 100.0 |
| SFA | 28.0 | 25.7 |
| MUFA | 38.9 | 53.2 |
| PUFA | 33.1 | 21.1 |
| LC-PUFA | 13.9 | 2.1 |
| n-6 C18 PUFA | 16.1 | 15.9 |
| n-6LC-PUFA | 1.0 | 0.3 |
| n-3 C18 PUFA | 3.1 | 3.1 |
| n-3 LC-PUFA | 12.9 | 1.8 |
