# Supplementary material for: The Expression of Pre- and Postcopulatory Sexually Selected Traits Reflects Levels of Dietary Stress in Guppies
Source: PLoS One. 2014 Aug 29;9(8):e105856. doi: 10.1371/journal.pone.0105856 (PMC4149491; doi:10.1371/journal.pone.0105856)
Supplement: Table S2 — Univariate models for all sexually selected traits. (DOCX) [file pone.0105856.s002.docx]

**Table S2.** Univariate models for all sexually selected traits.

| Response traits | Intake group  (*Mean±SE*) | | |  | | |  | n3LC group  (*Mean±SE*) | | | |  |  | Intake*n3LC | | | |
| --- | --- | --- | --- | --- | --- | --- | --- | --- | --- | --- | --- | --- | --- | --- | --- | --- | --- |
|  | High | Low | | Test statistic | | | P |  | Enriched | | Reduced | Test statistic | P |  | Test statistic | | P |
| *Precopulatory traits* |  | | | | | | | | | | | | | | | | |
|  |  |  | | | *Z* |  | |  | |  | | *z* |  | *z* | |  | |
| *Sexual interest* | 479.1±10.2 | 134.0±19.9 | | | -5.08 | ***<0.001*** | | 303.9±28.8 | | 309.2±26.1 | | 0.25 | 0.80 | -1.07 | | 0.28 | |
| *Sigmoid displays* | 8.4±0.72 | 0.88±0.32 | | | -6.83 | ***<0.001*** | | 4.75±0.73 | | 4.53±0.75 | | -0.01 | 0.99 | 2.01 | | ***0.044*** | |
| *Gonopodial thrusts* | 3.23±0.39 | 1.15±0.29 | | | -3.03 | ***0.002*** | | 2.07±0.35 | | 2.32±0.38 | | -0.33 | 0.74 | -0.09 | | 0.93 | |
|  |  |  | | | *F* |  | |  | |  | | *F* |  | *F* | |  | |
| *Orange area^* | 11.05±0.43 | 7.09±0.3 | | | 15.12 | ***<0.001*** | | 9.15±0.5 | | 8.96±0.4 | | 0.34 | 0.559 | 5.21 | | ***0.024*** | |
| *Orange spots* | 5.55±0.2 | 4.6±0.21 | | | 8.99 | ***0.003*** | | 4.98±0.2 | | 5.16±0.24 | | 0.25 | 0.62 | 0.003 | | 0.959 | |
| *Orange PC1* | 1.46±1.03 | -1.39±0.89 | | | 2.09 | 0.15 | | -0.75±0.9 | | 0.76±1.02 | | 1.10 | 0.296 | 0.13 | | 0.72 | |
| *Orange PC2* | 0.09±0.22 | -0.08±0.27 | | | 0.44 | 0.51 | | -0.29±0.2 | | 0.3±0.28 | | 2.79 | 0.097 | 0.007 | | 0.93 | |
| *Orange PC3* | 0.11±0.19 | -0.1±0.22 | | | 0.35 | 0.56 | | 0.15±0.2 | | -0.15±0.22 | | 0.99 | 0.32 | 0.18 | | 0.67 | |
| *Orange PC4* | 0.38±0.13 | -0.36±0.16 | | | 4.73 | ***0.032*** | | 0.11±0.1 | | -0.11±0.17 | | 1.42 | 0.24 | 1.51 | | 0.22 | |
| *Iridescent area^* | 9.26±0.42 | 5.82±0.21 | | | 4.37 | ***0.039*** | | 7.31±0.4 | | 7.75±0.45 | | 0.701 | 0.404 | 5.84 | | ***0.017*** | |
| *Iridescent spots^* | 7.81±0.27 | 6.22±0.22 | | | 2.55 | 0.113 | | 6.91±0.2 | | 7.11±0.29 | | 0.19 | 0.66 | 3.53 | | 0.063 | |
| *Iridescent PC1* | 2.43±0.94 | -2.31±0.8 | | | 5.05 | ***0.027*** | | -0.64±0.9 | | 0.65±0.97 | | 0.88 | 0.351 | 0.53 | | 0.470 | |
| *Iridescent PC2* | -0.49±0.43 | 0.47±0.36 | | | 0.04 | 0.85 | | -0.28±0.4 | | 0.28±0.37 | | 1.13 | 0.29 | 0.006 | | 0.94 | |
| *Iridescent PC3* | 0.14±0.27 | -0.14±0.3 | | | 0.35 | 0.56 | | -0.03±0.2 | | 0.03±0.34 | | 0.01 | 0.91 | 0.34 | | 0.56 | |
| *Postcopulatory traits* |  | | | | | | | | | | | | | | | | |
| *VAP* | 86.27±1.82 | | 74.47±2.1 | | 18.69 | ***<0.001*** | | 83.23±2.32 | | 78.23±1.85 | | 3.35 | 0.069 | 2.39 | | 0.125 | |
| *Sperm viability* | 0.77±0.02 | | 0.68±0.03 | | 7.42 | ***0.008*** | | 0.79±0.03 | | 0.67±0.02 | | 12.57 | ***<0.001*** | 1.68 | | 0.197 | |
| *Sperm number* | 136.6±3.09 | | 99.38±3.6 | | 61.99 | ***<0.001*** | | 122.7±4.3 | | 113.9±4.07 | | 2.92 | 0.091 | 0.06 | | 0.81 | |
| *Sperm length* | 54.59±0.12 | | 53.7±0.12 | | 30.66 | ***<0.001*** | | 54.02±0.14 | | 54.29±0.12 | | 3.001 | 0.086 | 1.12 | | 0.292 | |

Significant P-values are marked in bold and italic fonts.

Body length had a significant effect only for those traits marked with ^ (covariate effects: Orange area: F_1,110_= 4.79, P= 0.031; Iridescent area: F_1, 110_ = 30.14, P < 0.001; Iridescent spots: F_1, 110_ = 6.52, P = 0.012).
