# Supplementary material for: The Expression of Pre- and Postcopulatory Sexually Selected Traits Reflects Levels of Dietary Stress in Guppies
Source: PLoS One. 2014 Aug 29;9(8):e105856. doi: 10.1371/journal.pone.0105856 (PMC4149491; doi:10.1371/journal.pone.0105856)
Supplement: Table S3 — ANOVA results for fatty acids content (%) in body samples and testes tissues after the treatment. Significant P values are highlighted in bold and italic fonts. (DOCX) [file pone.0105856.s003.docx]

**Table S3.** ANOVA results for fatty acids content (%) in body samples and testes tissues after the treatment. Significant P values are highlighted in bold and italic fonts.

| Classes of FA | Body  (*Mean±SE*) | |  | | | Testes  (*Mean±SE*) | |  | |
| --- | --- | --- | --- | --- | --- | --- | --- | --- | --- |
|  | n3LC-enriched | n3LC-reduced | F | P |  | n3LC-enriched | n3LC-reduced | F | P |
| Saturated fatty acids (SFA) | | | | | | | | | |
| 14:0 | 2.27±0.05 | 1.7±0.08 | 35.39 | ***<0.001*** | | 1.01±0.17 | 0.78±0.13 | 1.07 | 0.31 |
| 16:0 | 22.14±0.2 | 21.79±0.3 | 0.87 | 0.354 | | 19.88±0.54 | 19.48±0.54 | 0.27 | 0.603 |
| 18:0 | 9.68±0.15 | 9.4±0.13 | 1.920 | 0.17 | | 13.02±0.45 | 12.9±0.51 | 0.03 | 0.86 |
| Monounsaturated fatty acids (MUFA) | | | | | | | | | |
| 16:1n-7 | 4.59±0.1 | 3.75±0.17 | 18.15 | ***<0.001*** | | 2.74±0.33 | 2.39±0.21 | 0.80 | 0.38 |
| 18:1n-9 | 28.16±0.4 | 32.3±0.56 | 35.22 | ***<0.001*** | | 19.36±1.19 | 23.11±1.25 | 4.75 | ***0.034*** |
| Polyunsaturated fatty acids (PUFA) | | | | | | | | | |
| n-6 Polyunsaturated fatty acids (n-6 PUFA) | | | | | | | | | |
| 18:2n-6 | 5.97±0.08 | 5.44±0.2 | 6.16 | ***0.016*** | | 5.72±1.18 | 4.44±0.17 | 1.06 | 0.307 |
| 18:3n-6 | 0.75±0.03 | 0.83±0.04 | 3.16 | 0.081 | | 0.23±0.07 | 0.29±0.07 | 0.34 | 0.562 |
| n-6 Long chian polyunsaturated fatty acids (n-6 LC-PUFA) | | | | | | | | | |
| 20:3n-6 | 0.45±0.07 | 1.52±1.07 | 0.99 | 0.323 | | 0.54±0.13 | 1±0.23 | 3.18 | 0.08 |
| 20:4n-6 | 1.41±0.08 | 1.51±0.07 | 0.77 | 0.384 | | 3.3±0.29 | 3.95±0.44 | 1.59 | 0.212 |
| 22:4n-6 | 0.67±0.04 | 0.67±0.04 | 0.00 | 0.989 | | 0.94±0.13 | 1.09±0.13 | 0.66 | 0.416 |
| 22:5n-6 | 0.42±0.03 | 0.45±0.03 | 0.88 | 0.353 | | 0.89±0.1 | 1.21±0.12 | 3.93 | 0.053 |
| n-3 Polyunsaturated fatty acids (n-3 PUFA) | | | | | | | | | |
| 18:3n-3 | 3.17±0.22 | 3.29±0.24 | 0.15 | 0.704 | | 1.15±0.22 | 1.25±0.17 | 0.15 | 0.695 |
| 18:4n-3 | 0.6±0.06 | 0.49±0.06 | 1.56 | 0.216 | | 0.22±0.06 | 0.2±0.05 | 0.05 | 0.818 |
| n-3 Long chain polyunsaturated fatty acids (n-3 LC-PUFA) | | | | | | | | | |
| 20:4n-3 | 0.49±0.06 | 0.51±0.06 | 0.11 | 0.745 | | 0.15±0.05 | 0.11±0.03 | 0.42 | 0.522 |
| 20:5n-3 | 1±0.06 | 0.61±0.05 | 25.07 | ***<0.001*** | | 0.69±0.08 | 0.29±0.07 | 15.10 | ***<0.001*** |
| 22:5n-3 | 1.8±0.05 | 1.09±0.06 | 87.89 | ***<0.001*** | | 1.82±0.09 | 1.09±0.11 | 27.08 | ***<0.001*** |
| 22:6n-3 | 6.33±0.19 | 4.9±0.22 | 23.27 | ***<0.001*** | | 18.35±1.41 | 16.89±1.22 | 0.60 | 0.441 |
| Fatty acid classes | | | | | | | | | |
| SFA | 34.83±0.3 | 33.65±0.5 | 4.28 | ***0.043*** | | 35.09±0.68 | 34.28±0.42 | 0.97 | 0.33 |
| MUFA | 40.22±0.4 | 42.38±0.8 | 5.68 | ***0.020*** | | 29.21±1.55 | 32.36±1.38 | 2.27 | 0.14 |
| PUFA | 24.95±0.4 | 23.97±1.2 | 0.58 | 0.448 | | 35.7±1.73 | 33.36±1.43 | 1.07 | 0.30 |
| n-6 PUFA | 11.32±0.2 | 12.01±0.7 | 0.83 | 0.365 | | 13.21±0.97 | 13.39±0.48 | 0.025 | 0.87 |
| n-6-LC PUFA | 4.61±0.26 | 5.74±0.9 | 1.37 | 0.247 | | 7.27±0.61 | 8.66±0.64 | 2.48 | 0.12 |
| n-3 PUFA | 13.63±0.3 | 11.96±0.6 | 6.08 | ***0.017*** | | 22.49±1.27 | 19.97±1.1 | 2.20 | 0.14 |
| n-3-LC PUFA | 9.86±0.22 | 8.17±0.6 | 6.94 | ***0.011*** | | 21.12±1.36 | 18.51±1.16 | 2.09 | 0.154 |
